# Is the hotel industry really committed to the environment? Answering using the business models framework

**DOI:** 10.1007/s11628-023-00522-2

**Published:** 2023-02-17

**Authors:** María A. Quintás, Ana I. Martínez-Senra, Adela García-Pintos

**Affiliations:** 1 ECOBAS, Universidade de Vigo, Department of Business Management and Marketing, Faculty of Economics, 36310, Vigo, Spain; 2 Universidade de Vigo, Department of Business Management and Marketing, Faculty of Social Sciences and Communication, 36005 Pontevedra, Spain

**Keywords:** Environmental Business Models, Hotel industry, Value proposition, Value creation, Value capture

## Abstract

This paper conceptualizes Environmental Business Models in the hotel industry as the result of a balanced emphasis on several initiatives regarding value proposition, value creation and value capture. It tests how this framework affects 120 Spanish hotel chains by assessing their sustainability reports and websites. The results show that Environmental Business Models are still poorly developed and present uneven progress in their components. This may be a sign that managers are currently using a partial perspective for environmental management with a focus on value creation initiatives that mainly have a marketing or a cost impact.

## Introduction

The COVID-19 crisis confirmed the negative impact of travel and tourism on the environment (O’Connor and Assaker 2021). For example, in Europe and China, CO_2_ emissions decreased by 17% due to the reduction in flights caused by the imposed restrictions. In addition, the decrease in tourists in some protected areas and beach destinations allowed the wildlife to rebound, improved water quality and reduced pollution (Rume and Islam 2020). Hotel chain (HC) managers have to regulate the effects that their HCs have on the environment, which is a source of attraction for potential customers, to avoid causing its deterioration.

Some of the most important problems that impact the environment around HCs are CO_2_ emissions, energy and water consumption and waste generation. The accommodation industry is responsible for 1% of CO_2_ emissions worldwide (UNWTO, 2017). Moreover, hotels spend 3–9% of their operational budget on energy (Becken 2013; Vourdoubas 2015), 4% of their total running cost on water (Deyà Tortella and Tirado 2011), and 2.3% of their annual turnover on food waste (Filimonau and De Coteau 2019), which represent the main types of waste in hotels. To adhere to the Paris Agreement, hotels will need to reduce their greenhouse gas emissions by 90% per room per year from 2010 up to 2050 (Perin 2017).

Traditionally, the literature on the hotel industry has only partially studied environmental problems. Some papers analyse the importance of corporate social responsibility actions (Levy and Park 2011), voluntary environmental tools such as ecolabels (Duglio et al. 2017; Gössling and Buckley 2016) and environmental management systems (EMSs) (Cavero-Rubio and Amorós-Martínez 2020; Chan and Wong 2006). Some of the literature also analyses how these actions affect economic performance (Segarra-Oña et al. 2012). Through these studies, we can undoubtedly learn not only about the reasons, barriers and facilitators behind adopting different environmental strategies, but also about the more integrative perspective that has been initiated through the Business Model (BM) framework.

BMs provide an integrated vision of the set of activities and processes carried out by firms when defining value proposition, creation and capture for their stakeholders (Bocken et al. 2014; Laasch 2018; Richardson 2008; Teece 2010). BM innovations are non-technical dimensions of the ‘disruptions’ in sustainability transitions (Kivimaa et al. 2021), which makes them a vital component of such transitions (Bocken and Short 2016; Sarasini and Linder 2018). Several authors have indicated that innovation in the BMs can speed up the process of decarbonisation (Mangelsdorf 2010; Wainstein and Bumpus 2016). These non-technological innovations can be relevant for service firms (Souto 2015), especially the hotel industry, where turnover is only positively related to complex innovation strategies (Martinez-Martinez et al. 2019). Nevertheless, studies on hotel BMs are recent and scarce, and pay very little attention to environmental issues.

For instance, Reinhold et al. (2017) reviewed 28 papers that made explicit use of the BM concept in tourism, including 14 on tourism and hospitality management. Only 2 of them addressed the environmental effects of the hotel industry (Høgevold et al. 2015; Mihalič et al. 2012). Mihalič et al. (2012) analysed Slovenian hotels’ sustainable BMs, and their results indicate that great importance is placed on economic and marketing indicators, but very little attention is paid to the environmental indicators. Høgevold et al. (2015) assessed the feasibility of a sustainable manufacturing BM of a major Scandinavian HC. Furthermore, Coles et al. (2017) found a predominant belief that guests have no interest in environment issues and that HCs gear their BMs toward increasing income. However, the authors consider it necessary to conduct more studies in the tourism sector with Environmental BMs (EBMs) as the unit of analysis to understand how firms value their environmental cost and resources.

There is great room for improvement in researchers’ understanding of how BM design can help implement a systematic environmental commitment. This paper seeks to conceptualize EBM in the hotel industry as a mix of particular initiatives with regard to environmental value proposition, creation and capture. This investigation uses evidence from 120 top Spanish HCs in order to study the level of EBM implementation and to identify the strengths and weaknesses in the industry.

The paper is structured in the following way. Section 2 analyses how HCs integrate environmental issues into their BMs. Section 3 describes the sample and methodology used. Section 4 includes the results and discussion. Finally, Sect. 5 contains the conclusions and implications for business management.

## Literature review

### Business Models (BMs) and hotels’ environmental problems

Researchers have developed three possible interpretations for BMs*:* attributes of a real firm, cognitive or linguistic outline and conceptual representation (Massa et al. 2017; Reinhold et al. 2017)*.* This study uses the most specific concept possible, which is the attributes of a real firm. In this interpretation, BMs are considered a set of activities and processes carried out by firms to define value proposition, creation and capture for their stakeholders (Bocken et al. 2014; Bocken and Short 2016; Richardson 2008).

However, as sustainability concerns spread throughout customer bases and regulations, the conceptualization of BMs has increasingly incorporated an environmental perspective. This is particularly important in the hotel sector, where environmental impact is considered a competitive priority (Espino-Rodríguez 2016). Energy and water consumption and waste generation incur great costs for hotels (Becken 2013; Deyà et al. 2011; Filimonau and De Coteau 2019), an issue that may be an opportunity for them because “green” customers are on the rise (Preziosi et al. 2019). The literature has already identified BMs focused exclusively on environmental aspects to deliver implicitly or explicitly what can currently be included within the category of EBMs. Some examples include BMs focused on maximising material and energy efficiency, creating value from waste or closing resource loops and substituting them with renewables and natural processes (Bocken et al. 2014; Bocken et al. 2019).

This study reviews the environmental and BM literature to identify which key elements should be included in the BMs of environmentally committed HCs. Following Bocken et al. (2014), we extrapolate the main components of a BM (Richardson 2008) from an EBM to distinguish between environmental value proposition, environmental value creation and delivery and environmental value capture.

### Environmental value proposition

The value proposition is one of the most important factors for BM success in the hotel industry (Langvinienė and Daunoravičiūtė 2015). It shapes the functional and emotional outcomes of travel experiences and influences the evaluation of service experiences (Li et al. 2021). Value proposition is related to a firm’s strategic positioning (Richardson 2008) and its organisational orientation (Laasch 2018). It defines what will be delivered to its customers, what characteristics customers will be prepared to pay for, and what the firm’s basic approach to obtaining competitive advantages will be (Richardson 2008). Value proposition in an EBM should provide measurable ecological and social value in relation to its economic value (Boons and Lüdeke-Freund 2013; Schaltegger et al. 2016). The review of the literature allowed the following key elements of an environmental value proposition to be identified:

#### Stakeholders’ environmental needs

The value proposition in a conventional BM used to focus on satisfying the needs of customers (Teece 2010) who, in many hotel managers’ opinion, had no interest in green credentials (Coles et al. 2017). However, nowadays, customers increasingly value environmental issues when travelling (Bohdanowicz and Zientara 2008) and are more sensitive to information about the environment (Preziosi et al. 2019). Moreover, the value proposition in an EBM has to consider the needs of other stakeholders (e.g. investors, shareholders, business partners, employees and suppliers) (Freudenreich et al. 2019; Velter et al. 2020) because these can be key to corporate sustainability by stressing certain corporate goals and adopting win–win environmental solutions (Morioka et al. 2016). Therefore, the environmental value proposition must combine all of the stakeholders’ needs into a more meaningful and enriching value proposition (Baldassarre et al. 2017).

#### Mission, vision and objectives

Stubbs and Cocklin (2008) highlight in their conceptualization of a sustainable BM that it is important to include environmental aspects when defining an organisation’s purpose and considering all of the stakeholders’ needs. Vision development should identify stakeholders’ values and incorporate them into a strategy that determines environmental targets (Wagner and Svensson 2014). Moreover, shared visions allow resources to be integrated and managed adequately to respond to customers’ dynamic needs (Tang et al. 2015).

Including environmental aspects in an HC’s mission and vision has a strong predictive power to explain visitors’ stay preference, service quality, willingness to pay and brand image (Kucukusta et al. 2013). In recent years, the number of hotels that have incorporated social and environmental objectives into their missions has been increasing (Chung and Parker 2010; Kucukusta et al. 2013), although this is still not the most widely used practice (Holcomb et al. 2007).

#### Environmentally friendly service offer

A value proposition is typically concerned with the product and service offered to generate economic returns (Bocken et al. 2014; Osterwalder et al. 2005). In an EBM, environmental products or service offers that consider the needs of stakeholders are a key element of value proposition (Baldassarre et al. 2017). This includes products or services that use fewer resources, generate less waste and emissions and create less pollution than other products or services that deliver similar functionality (Bocken et al. 2014).

### Environmental value creation

To create and deliver value, it is necessary to establish how the firm will compete to put its value proposition into practice (Morioka et al. 2016; Richardson 2008). This is the core of any BM (Bocken et al. 2014) and includes the resources, activities, key processes, and links with suppliers, partners and clients that will allow the value proposition to be implemented (Bocken et al. 2014; Richardson 2008). When a value’s creation and delivery are designed, the value proposition and capture must be kept in mind (Richardson 2008). Value creation in EBMs cannot be achieved through activities that damage the environment. Therefore, the literature was reviewed to find the following key elements of environmental value creation:

#### Environmental management

An EBM should incorporate organisational activities, processes and practices that allow it to reach excellence when managing environmental issues (Laasch 2018). Such activities include operational practices that solve the HC’s main environmental problems, including those focused on achieving energy efficiency and reducing CO_2_ emissions, water consumption, waste generation or noise pollution (Hsieh 2012; Milanés-Montero et al. 2014).

These operational practices may be carried out alone or in association with other organisational practices, such as ecolabels and/or EMSs. Both types of certifications are increasingly being applied in HCs (Cavero-Rubio and Amorós-Martínez 2020). Ecolabels certify a company’s environmental performance in certain areas and provide information to consumers to influence their behaviour towards the environment (Gössling and Buckley 2016). More than a hundred ecolabels are applied in tourism (Gössling and Buckley 2016), including some prominent examples in the hotel industry such as the European ecolabel, Green Globe 21 and Travelife. EMSs manage companies’ environmental performance and continuously improve it according to a planned strategy. The most important EMSs in the hotel industry are ISO 14001 (International Standard) and EMAS (European Regulation) (Cavero-Rubio and Amorós-Martínez 2020; Ouyang et al. 2019).

#### Partners and contact networks

The value created and delivered in an EBM is highly complex and involves multi-relational systemic interactions that emphasize the roles of the different stakeholders (Laasch 2018; Velter et al. 2020), which employees and customers stand out from (Chou et al. 2018; Del Brío et al. 2007).

Employees are fundamental in an EBM because they link the tourism service offered (value proposition) with the value perceived by customers (O’Cass and Sok 2015). Moreover, many environmental practices need eco-friendly behaviour from employees, which requires appropriate human resource management to improve employees’ environmental knowledge, awareness and behaviour (Chan et al. 2017; Kim et al. 2019).

Customers are another key agent that create value for hotels. The firm may offer a value proposition, but value realization depends on customers’ participation in the service process. Environmental education (Scanlon 2007) and co-creation between firms and customers facilitate such participation (Cabiddu et al. 2013; Lee and Kim 2019; Su et al. 2016; Yen et al. 2021). Another technique that allows other relevant hotel stakeholders to participate is co-production, which facilitates the integration and application of resources contributed by both suppliers and consumers (Chathoth et al. 2013).

Finally, other agents, such as on-line travel agencies, environmental organisations or NGOs, are important too. EBMs should align the interests of all stakeholder groups in value creation (Bocken et al. 2014; Velter et al. 2020).

### Environmental value capture

Value capture focuses on analysing what benefits companies obtain from participants and how they obtain them, since value proposition and creation do not guarantee business success if companies cannot appropriate the value (Richardson 2008). Therefore, the relationship between the dimensions of value creation and value capture is key in BMs (Amit and Zott 2001). Traditional literature on BMs points out that value capture describes how a firm generates revenue and profit (Richardson 2008). However, the literature on environmental and sustainable BMs includes other forms of non-monetary value capture, such as lowering environmental footprint or reducing synthetic waste in landfills (Bocken et al. 2014; Morioka et al. 2017). Therefore, it will also be necessary to assess whether the environmental techniques implemented (environmental value creation) to carry out the environmental value proposal manage to reduce the company’s environmental impact. Notably, there are companies that only seek symbolic environmental legitimacy and carry out environmental management practices that fail to reduce their environmental impact (Quintás and Martínez-Senra 2022).

The literature reviewed identified the following key elements in environmental value capture:

#### Revenue model and cost structure

Value capture in EBMs has to be viable in economic and environmental terms (Morioka et al. 2017). This includes considering the monetary consequences of the means employed in the BM (cost structure) and the way a company makes money through a variety of revenue flows (revenue model) (Osterwalder et al. 2005). Hotels with EBMs may report better profits on both the revenue side by charging a price premium (Zhang et al. 2020) and the cost side (Coles et al. 2017). Financial performance is an enabler that is used to enhance environmental performance and is important to relevant stakeholders such as shareholders (Morioka et al. 2017). The prospects of economic savings and increasing customer demand are crucial to the hotel industry’s environmental awareness and responsible environmental management (Bohdanowicz 2005).

#### Non-financial value

EBMs also capture non-financial value, such as employees feeling proud, motivated, and aligned with the company’s values or client’s feeling satisfied after using more environmental products (Morioka et al. 2017).

Another way to capture non-financial value is through a company’s contribution to environmental improvement (Bocken et al. 2014). The literature frequently identifies CO_2_ emissions, energy, water and electricity consumption and waste generation when studying HCs’ environmental impact (Baxter and Srisaeng 2021; Jones and Wynn 2019). These consumption and emissions represent a significant cost in the budgets of HCs (Becken 2013; Deyà Tortella and Tirado 2011; Filimonau and De Coteau 2019; Vourdoubas 2015).

## Methods

To determine if hotels incorporate the key elements identified in the literature review in each of the EBM components (i.e. value proposition, creation and capture), we performed a content analysis of the sustainability reports and websites of the main Spanish HCs. All reports that include information on environmental impact were considered, including CSR reports, sustainability reports, environmental statements and annual reports, among others. The on-line information search was carried out between April and May 2019. It is worth noting that, according to the UNWTO tourism dashboard (UNWTO n.d.), Spain was the world’s top tourist destination in 2019 based on arrivals of tourists (83.7 million). In 2019, the Spanish tourist industry included 14,897 hotels and generated 2.6 million jobs. The most recent available data shows that it represented 12.7% of GDP in 2018 (INE 2017). Therefore, it would not be strange to find 4 Spanish multinationals among the world’s 50 largest HCs (Meliá, Barceló, Riu and Iberostar), according to the ranking in the American Hotels Magazine (Weinstein 2020).

### Sample

The initial population of hotels in this study is comprised of 120 Spanish HCs with more than 1,000 rooms, which were collected from the Hosteltur ranking (Hinojosa 2018). Of these 120 HCs, 29 had websites and a sustainability report or equivalent, and 24 had websites with information on environmental aspects. A request email was sent to the HCs that we found no published environmental information for, which allowed us to obtain one more HC environmental report. The final sample was comprised of 54 HCs (see HCs in Tables 3–9); in other words, 45% of the population (120 HCs), about the same percentage that other studies have often achieved (e.g. 46% in Hsieh 2012).

This study only focuses on the largest HCs for two reasons. First, chain-affiliated hotels are generally more active in environmental issues than individually owned-and-managed facilities (Bohdanowicz 2005; Milanés-Montero et al. 2014) because they are larger and have more financial resources and a better-known brand image, which they need to preserve. Second, non-chain hotels are less engaged in the dissemination of environmental information online (Hsieh 2012).

### Content analysis

The use of content analysis has grown over time in tourism studies (Camprubí and Coromina 2016; Hall and Valentin 2005). This study performed a content analysis of the sustainability reports or equivalents and websites of the total sample of Spanish HCs. Such reports and websites are the main channels used by firms to communicate their commitment to environmental sustainability (Bonilla-Priego and Benítez-Hernández 2017; Paul 2008). This method allowed the researchers to examine the documents and websites for content and to make replicable and valid inferences from information found in the text of each document or website, along with the contexts in which the information was used. It also enabled them to gain greater understanding of the specific phenomenon (EBMs) (Krippendorff 2004).

The advantages of content analysis are numerous; however, the main drawback is the potential influence of the researcher (Hall and Valentin 2005). Researcher bias can potentially constrain decisions in data collection, analysis and interpretation in favour of the research hypothesis. Therefore, a restrictive criteria to code all items was employed; if HCs’ public information only described a declaration of intent without concrete practices or initiatives, we coded them with a 0. Moreover, two independent researchers read and coded the information collected from the reports and/or websites. The reliability of the coding procedure was evaluated by calculating the agreement ratio; if it was greater than 96%, it was considered satisfactory (Beattie et al. 2004). All authors of this paper met to jointly evaluate the few cases in which there was no agreement on coding and, in the case of doubts about the presence or absence of any of the items, to apply a restrictive criterion that considered the non-presence of an item.

Table 1 lists the coding criteria for the key elements of the EBM components and Annex 1 presents some coding examples for each of the EBM components. The environmental value proposition component can be assigned values between 0 and 17 depending on whether the 17 items described in the literature review that relate to the three key elements are present or not.

**Table 1:** Coding criteria

Proposition (0,17)	Creation (0,12)	Capture
*Identification of stakeholders’ needs (0,10)* Customers (0,1) Suppliers (0,1) Employees (0,1) Administrators (0,1) Shareholders (0,1) Society (0,1) Partners & owners (0,1) Press & media (0,1) Professional associations (0,1) Stakeholders in general (0,1) *Definition of mission, vision and objectives (0,3)* Mission (0,1) Vision (0,1) Objectives (0,1) *Definition of environmentally friendly service offers (0,4)* Accommodation (0,1) Water (0,1) Food (0,1) Transport (0,1)	*Number of initiatives (0,6)* Energy efficiency (0,1) CO_2_ emissions (0,1) Water conservation (0,1) Waste generation (0,1) Biodiversity protection (0,1) Noise (0,1) *Presence of EMS (0,1)* ISO 14001 (0,1) EMAS (0,1) ISO 50001 (0,1) *Presence of Ecolabels (0, 1)* European ecolabel (0,1) Travelife (0,1) Biosphere (0,1) Green Globe (0,1) Alcudia ecotourism label (0,1) Hotels + Verdes (0,1) Earthcheck (0,1) Green Key (0,1) Leed (0,1) Sapling (0,1) BREEAM (0,1) Marti (0,1) Generalitat environmental quality label (0,1) CPD (0,1) Green Leaders (0,1) *Number of partners (0,4)* Suppliers (0,1) Customers (0,1) Employees (0,1) Environmental organisations (0,1)	*Non-financial value* CO_2_ emissions (0,1,2,3) Energy consumption (0,1,2,3) Water consumption (0,1,2,3) Electricity consumption (0,1,2,3) Waste generation (0,1, 2,3)

The first key element (stakeholders’ needs) can receive values between 0 and 10. Each stakeholder was coded with a 1 when the HC took its needs into account, regardless of its importance in the strategy of each HC, since this information is not available in the reports. Otherwise, it was coded with a 0.

The second key element (mission, vision and objectives) can receive values between 0 and 3. Each of the 3 items was coded with a 1 if the environmental aspects were taken into account in their definition and with a 0 otherwise.

Finally, the third key element (environmentally friendly service offers) can receive values between 0 and 4. Each of the 4 items (accommodation, water, food and transport) was coded with a 1 if the HCs’ offer was respectful of the environment and with a 0 otherwise.

The value creation component can be assigned values between 0 and 12 depending on whether the items identified in relation to its two key elements are present or not. The first element (resources, processes, and practices carried out in environmental management) was grouped into 3 types (see Table 1): 1) Initiatives launched to reduce environmental impact, which can receive values between 0 and 6 depending on the initiatives carried out. Each initiative was coded with a 1 if the HC performed it and with a 0 otherwise. 2) The presence of EMSs, which can receive values between 0 and 1. This was coded with a 1 if the HC had any of three analysed EMSs and with a 0 otherwise. 3) The presence of ecological labels, which can receive values between 0 and 1. This was coded with a 1 if the chain presented any of the analysed labels in a hotel and with a 0 otherwise.

The second key element (HC partners for managing environmental problems) can be assigned values between 0 and 4 depending on how many partners collaborate with the HC. In this case, each partner was coded with a 1 if they collaborated with the HC, regardless of its importance to the strategy of each HC, since this information is not available in the reports. Otherwise, it received a 0.

For the environmental value capture component, two key elements were identified: financial and non-financial value. In both cases, the HCs publish very little information. In regards to the first element (revenue model and cost structure), most of the HCs do not report on their websites or in their environmental reports about how their cost and revenue structures are affected by their environmental actions. In regards to the second element (non-financial value), issues such as employee loyalty and customer retention are not mentioned in public information. Therefore, these types of data have not been included in the content analysis.

Value capture is made up of 5 non-financial items that reflect HCs’ environmental impact: CO_2_ emissions, energy, water and electricity consumption and waste generation. Each item was coded with a 0 if they did not include these indicators, a 1 if there was an increase, a 2 if there was no change and a 3 if there was a reduction. To codify these items, it is necessary that the HCs include the last 2 years in their reports or websites.

Finally, the HCs’ date of creation was included to calculate their age and the number of hotels and beds was included to quantify their size.

## Results

Table 2 presents the frequency of items in the HCs’ EBM components. Regarding the environmental value proposition component, the environmental objectives setting bears the greatest weight (61.1%), whereas the environmental aspects are not included in the mission or vision. Only 9 HCs integrate environmental issues simultaneously in their mission, vision and objectives: Iberostar, Princess, Paradores, Vincci, Med Playa, Seaside, Fuerte, Coral and Nuñez i Navarro. This low level of environmental aspect integration in firms’ missions and visions was already detected over 10 years ago in studies such as Holcomb et al. (2007). Furthermore, this is consistent with the fact that most of the HCs apparently neglect stakeholders’ needs. Customers, employees and suppliers’ needs are only considered by 16, 12 and 11 HCs, respectively; only 8 HCs take the needs of 5 or more stakeholder groups into account when defining their environmental value proposition (Meliá, NH, Bahia Principe, Lopesan, Portaventura, Fuerte, Coral and Gloria Thalasso).

**Table 2:** Items in hotel chains’ environmental business model components

Proposition			Creation			Capture		
Key elements	No.	%	Key elements	No.	%	Key elements	No.	%
*Identification of stakeholders’ needs*			*No. of initiatives*			*CO*_2_* emissions*		
Customers	16	29.6	Energy efficiency	45	83.3	Not specified	34	63
Suppliers	11	20.4	CO_2_ emissions	40	74.1	Increase	4	7.4
Employees	12	22.2	Water conservation	38	70.4	No change	0	0
Administrators	7	13	Waste generation	43	79.6	Reduction	16	29.6
Shareholders	5	9.3	Biodiversity protection	25	46.3	*Energy consumption*		
Society	8	14.8	Noise	11	20.4	Not specified	30	55.5
Partners & owners	2	3.7	*Presence of environmental management systems*			Increase	9	16.70
Press and media	2	3.7	ISO 14001	25	46.3	No change	0	0
Professional associations	3	5.6	EMAS	10	18.5	Reduction	15	27.8
Stakeholders in general	6	11.1	ISO 50001	3	5.6	*Water consumption*		
*Definition of mission, vision and objectives*			*Presence of ecolabels*			Not specified	27	50
Mission	13	24.1	European ecolabel	2	3.7	Increase	14	25.9
Vision	14	25.9	Travelife	31	57.4	No change	0	0
Objectives	33	61.1	Biosphere	3	5.6	Reduction	13	24.1
*Definition of environmentally-friendly service offers*			Green Globe	3	5.6	*Electricity consumption*		
Accomodation	7	13	Alcudia ecotourism label	2	3.7	Not specified	33	61.1
Water	0	0	Hoteles + Verdes	1	1.9	Increase	11	20.4
Food	5	9.3	Earthcheck	4	7.4	No change	0	0
Transport	10	18.5	Green Key	2	3.7	Reduction	10	18.5
			Leed	2	3.7	*Waste generation*		
			Sapling	1	1.9	Not specified	42	77.8
			BREEAM	1	1.9	Increase	5	9.2
			Marti	4	7.4	No change	0	0
			Generalitat environmental quality label	4	7.4	Reduction	7	13
			CPD	2	3.7			
			Green Leaders	7	1.3			
			*No. of partners*					
			Suppliers	35	64.8			
			Customers	34	63			
			Employees	37	68.5			
			Environmental organisations	21	38.9			

The more detailed analysis also suggests that environmentally friendly service offers are the least defined key element of the HCs’ environmental value proposal. In fact, no HC has an environmental offer in relation to water services, and only 3 hotels (Meliá, NH and Garden) have service offers that cover accommodation, food and ecological transport. Therefore, the capacity to generate environmental value services in order to attract green clients is apparently one of the challenges faced by the hospitality sector when drawing up EBMs.

In contrast with value proposition, it is worth noting that value creation is the most developed EBM component. The resources, processes and practices related to environmental management are the elements that carry most of the weight. A high percentage of hotels create initiatives to improve energy efficiency (83.3%) and to reduce waste generation (79.6%), CO_2_ emissions (74.1%) and water consumption (70.4%). These percentages are lower in the case of biodiversity protection (46.3%) and noise reduction (20.4%). Moreover, we identified 8 HCs that carry out these six initiatives: Bluebay, Princess, Grupotel, Garden, Portaventura, Fuerte, Gloria Thalasso and Portblue. However, in the analysis of the third BM component, these environmental practices do not seem to translate into environmental capture, or, at least, the HCs do not report on their environmental performance. In the marketing arena, 38 HCs (70.4%) have some type of ecolabel. Travelife is the most common ecolabel and is held by 31 HCs. The other certifications are used much less. Only the following 6 HCs have more than two certifications: Meliá (7), NH (6), Iberostar (5), H10 (5), Catalonia (4) and Sandos & Marconfort (3). In any case, ecolabels are used more than EMSs, which can potentially be explained by the higher cost and environmental commitment that the latter can entail. Only 25 HCs hold an ISO 14001 certification, 10 HCs present an EMAS and 3 have earned an ISO 50001. Gloria Thalasso holds 3 EMS certificates, whereas 8 HCs (i.e. Iberostar, Princess, Garden, Portaventura, RH, GF, Viva and Portblue) hold ISO 14001 and EMAS.

Regarding cooperation and contact networks for managing environmental issues, it is interesting that most of the chains collaborate with different types of partners. Moreover, the percentage of collaboration in this particular component of BMs is higher than the percentage found for the consideration of stakeholders’ needs in the analysis of the value proposition. HCs collaborate mainly with employees (68.5%), followed by suppliers (64.8%) and customers (63%). Although these percentages are not very low, HCs should probably achieve greater collaboration with: (1) customers who use resources in their establishments and whose consumption often shows non-sustainable behavioural patterns (Barr et al. 2011); and (2) employees who play a critical role in the successful creation and delivery of services to customers as those services are created during employee-customer interaction (Melton and Hartline 2010; O’Cass and Sok 2015).

Finally, environmental value capture is the least developed BM component of all three. Most of the HCs do not specify any of the indicators analysed in their reports and websites. The most widely used indicators are water consumption (50%) and energy consumption (44.5%), while the least reported are waste generation (22.2%) and CO_2_ emissions (37%). This low level of environmental performance is aligned with previous literature such as Mihalič et al. (2012), who found that only 28% of the hotels reported environmental performance. It is also coherent with De Grosbois (2012), who showed that HCs reported their environmental performance much less than environmental practices or goals.

The following tables (3 to 9) show the HCs grouped according to the degree of development in their EBM. This analysis allows the identification of the HCs that present more developed EBMs. To make the HC groups, we took into account the average values of the components of value proposition (2.8) and value creation (7.27) and whether they reduce emissions or consumption by more than half of the value capture indicators. Thus, we consider HCs to have a good level of development for their components if they present values equal to or greater than average (3 in the value proposition and 7 in the value creation) and if they reduce emissions or consumption by three or more value capture indicators. Applying these criteria we have identified seven different groups of HCs.

The first group consists of nine HCs that have a well-developed EBM and that reduce emissions, consumption or waste in most environmental capture indicators relevant to HC (Table 3). These HCs (Nuñez i Navarro, H10, Gloria Thalasso, Meliá, Bahía Principe, Portblue, NH, Mac and Vincci) are the best reference to other HCs that want to improve their EBM.

**Table 3:** Hotel chains with well-developed Environmental Business Models (proposition, creation and capture)

	Hotels	Beds	Age	Mission, vision…	Stakeh. needs	Services offered	Total propos	Breadth of initiatives	EMS	Ecolabel	Partner number	Total creation	CO_2_	Energy	Water	Electricity	Waste
**Nuñez i Navarro**^**2**^	12	1117	19	3	4		**7**	3	1		3	**7**	3	3	3	3	
H10	60	15,975	40		3		**3**	5		1	3	**9**	3	3	3		3
**Gloria Thalasso**^**1**^	4	1145	32	2	7		**9**	6	1	1	3	**11**	3	3	1	3	3
**Meliá**	321	80,861	34	2	8	3	**13**	5	1	1	4	**11**	3	1	3	3	1
**Bahía Principe**^**2**^	27	13,627	25	1	6		**7**	5		1	4	**10**	3	3	1	3	
**Portblue**^**1**^	8	1135	39	1	2		**3**	6	1	1	4	**12**	3	3	1	1	3
**NH**	385	59,682	32	1	5	3	**9**	5	1	1	3	**10**	3	3	3		
**Mac**	5	1682	37	1	3	1	**5**	4		1	2	**7**	3		3	3	
**Vincci**	39	5663	19	3	1	1	**5**	5	1	1	3	**10**		3	3	3	
**Average**	**95.67**	**20,098.56**	**30.78**				**6.78**					**9.67**					

Hotel chains in bold had environmental information on their websites and environmental reports, while the rest only had websites. (1) Information on the hotels in the chain; (2) For information about the business group, we only used data from the hotel chains

The second group consists of eight HCs (Grupotel, Fuerte, Lopesan, Garden, Coral, Med Playa, Princess and Portaventura) with well-developed EBMs in relation to value proposition and creation, but they need to improve their value capture (Table 4). They account for more than half of the value capture indicators, but most of them increase their emissions, consumption or waste. These HCs are on the right track, since the first step to achieving effective reductions in value capture indicators is to collect information on them.

**Table 4:** Hotel chains with Environmental Business Models that need improvement in the capture value

	Hotels	Beds	Age	Mission, vision…	Stakeh. needs	Services offered	Total propos	Breadth of initiatives	EMS	Ecolabel	Partner number	Total creation	CO_2_	Energy	Water	Electricity	Waste
**Grupotel**^1^	35	6267	34	1	3		**4**	6	1	1	4	**12**	1	1	3	3	1
**Fuerte**	6	1387	63	3	6	2	**11**	6	1	1	4	**12**	3	1	1	3	
**Lopesan**	20	7463	48	2	5	1	**8**	4	1	1	1	**7**	3	3	1	1	1
**Garden**^1^	14	2863	27	1	2	3	**6**	6	1	1	4	**12**	1	3	3	1	1
**Coral**	8	1243	34	3	5		**8**	4		1	3	**8**		3	1	1	1
**Med Playa**	16	3888	53	3			**3**	5	1	1	3	**10**	3	1	1	1	
**Princess**^1^	23	9952	53	3	3		**6**	6	1	1	4	**12**	3	1	1		
**Portaventura**^2^	5	2078	18	1	5		**6**	6	1	1	3	**11**		1	1	1	
**Average**	**15.88**	**4392.63**	**41.25**				**6.50**					**10.50**	**1.75**	**1.75**	**1.50**	**1.38**	**0.50**

All hotel chains had environmental information on their websites and environmental reports, while the rest only had websites. (1) Information on the hotels in the hotel chain. (2) For information about the business group, we only use hotel chain data

The third group of HCs is also on the right track. This group consists of three HCs (Riu, GF and Hovima) that have well-developed EBMs in relation to value creation and capture, but they need to improve their value propositions (Table 5). Although they address environmental issues in their missions, visions or objectives, they can improve their analysis of their stakeholders’ environmental needs and their environmentally friendly service offerings.

**Table 5:** Hotel chains with Environmental Business Models that need to improve their value propositions

	Hotels	Beds	Age	Mission, vision…	Stakeh. needs	Services offered	Total propos	Breadth of initiatives	EMS	Ecolabel	Partner number	Total creation	CO_2_	Energy	Water	Electricity	Waste
**Riu**	90	42,497	43	1			**1**	5		1	4	**10**	3	3	3		3
**GF**^1^	5	1603	49	1			**1**	5	1		3	**9**	3	3	3	1	3
**Hovima**^1^	7	1708	33	1			**1**	4		1	4	**9**			3	3	3
Average	**34,00**	**15,269,33**	**41,67**				**1.00**					**9.33**					

All hotel chains had environmental information on their websites and environmental reports. (1) Information on the hotels in the chain

The fourth group consists of seven HCs (Inturotel, Aqua & Ohla, Insotel, Hipotels, Sirenis, Senator and Be Live2) with an EBM for which they need to improve the value proposition and capture (Table 6). They neglect the key elements of value proposition (discussed above), and although they account for some value capture indicators, most of them do not reduce their emissions, consumption or waste.

**Table 6:** Hotel chains with Environmental Business Models that need to improve their value propositions and capture

	Hotels	Beds	Age	Mission, vision…	Stakeh. needs	Services offered	Total propos	Breadth of initiatives	EMS	Ecolabel	Partner number	Total creation	CO_2_	Energy	Water	Electricity	Waste
**Inturotel**	7	1107	33	1			**1**	4		1	2	**7**		1	3	1	3
Aqua & Ohla	9	1900	34	2			**2**	4		1	4	**9**		3	3		
**Insotel**	7	2412	15	1		1	**2**	4	1	1	4	**10**	1	1	1	3	
Hipotels	29	6483	24		1		**1**	4	1		4	**9**	1	3	1	1	
**Sirenis**	11	3838	50	1		1	**2**	5	1	1	4	**11**		3	1	1	
Senator	35	7858	53	1		1	**2**	5		1	3	**9**	3				
**Be Live**^**2**^	32	9108	18	1	1		**2**	3	1	1	2	**7**		1	1	1	
**Average**	**18,57**	**4672,29**	**32,43**				**1.71**					**8.86**					

Hotel chains in bold had environmental information on their websites and environmental reports, while the rest only had websites. (2) For information about the business group, we only use data from the hotel chains

The fifth group consists of four HCs (Iberostar, Paradores, Protur and Seaside) without any information on environmental value capture (Table 7). This group seems to have a higher preoccupation with environmental issues. They have well-developed value proposition and creation, but they do not report any information about their value capture. This behaviour can be associated with an environmentally symbolic compromise more than a substantive compromise, since the manager does not have an interest in knowing whether or not there is a reduction in the environmental impact of the HCs.

**Table 7:** Hotel chain without information about environmental value capture

	Hotels	Beds	Age	Mission, vision…	Stakeh. needs	Services offered	Total propos	Breadth of initiatives	EMS	Ecolabel	Partner number	Total creation	CO_2_	Energy	Water	Electricity	Waste
Iberostar	100	31,824	70	3		1	**4**	4	1	1	2	**8**					
**Paradores**	97	6125	29	3		1	**4**	5	1		4	**10**					
Protur	17	3561	35	1	1	1	**3**	5		1	1	**7**					
**Seaside**^**1**^	11	2175	36	3			**3**	5		1	2	**8**					
** Average**	**56,25**	**10,921,25**	**42,50**				**3.50**					**8.25**					

Hotel chains in bold had environmental information on their websites and environmental reports, while the rest only had websites. (1) Information on the hotels in the chain

The sixth group consists of seven HCs (Globales, Nordotel, RH, Be Cordial, Dunas, Catalonia and Bluebay) with poorly developed EBMs (Table 8). Their EBMs are oriented towards value creation, without any information about environmental value capture and limited environmental value propositions. As in the previous group, this profile can also be associated with behaviours seeking symbolic environmental legitimacy.

**Table 8:** HCs with EBMs oriented towards value creation without information about environmental value capture and a poor environmental value proposition

	Hotels	Beds	Age	Mission, vision…	Stakeh. needs	Services offered	Total propos	Breadth of initiatives	EMS	Ecolabel	Partner number	Total creation	CO_2_	Energy	Water	Electricity	Waste
Globales	40	8750	50	1			**1**	5		1	4	**10**					
Nordotel	17	4842	32		1		**1**	3	1		3	**7**					
RH	16	1884	40	1			**1**	4	1		3	**8**					
**Be Cordial**	9	1731	16	1			**1**	5		1	2	**8**					
Dunas	4	1192	31	1			**1**	5	1		3	**9**					
**Catalonia**	69	10,150	37					5		1	4	**10**					
Bluebay	56	11,230	57					6		1	3	**10**					
**Average**	**30,14**	**5682,71**	**37,57**				**0.71**					**8.86**					

Hotel chains in bold had environmental information on their websites and environmental reports, while the rest only had websites

Finally, the seventh group of HCs consists of the highest number of HCs with 16: Ilunion, Servatur, Oca, Sandos & M., Best, PY, Spring, Viva, Selenta, Serhs, Universal, Poseidon, Ibersol, AC, Santos and Blau. These firms lack EBMs (Table 9). Their BMs are not oriented towards environmental improvement in any components of their EBMs (value proposition, creation or capture).

**Table 9:** HCs with BMs not oriented towards environmental improvement (without key elements in environmental value proposition, creation and capture)

	Hotels	Beds	Age	Mission, vision…	Stakeh. needs	Services offered	Total propos	Breadth of initiatives	EMS	Ecolabel	Partner number	Total creation	CO_2_	Energy	Water	Electricity	Waste
**Ilunion**^**2**^	26	4137	27	1		1	**2**	4		1	1	**6**	3		1		
Servatur	8	1744	44	1			**1**	2			3	**5**					
Oca	25	1332	23	1			**1**	2			1	**3**					
Sandos & M	12	4508	18	0		1	**1**	3		1		**4**					
Best	33	10,968	26	1			**1**										
PY	6	1101	5	1			**1**										
Spring	3	1076	35					4	1	1		**6**					
Viva	7	1387	22					2	1	1		**4**					
Selenta	6	2900	16					2			1	**3**					
**Serhs**^**2**^	9	1130	45						1	1		**2**					
Universal	16	1992	57								1	**1**					
Poseidon	8	1770	42					1				**1**					
**Ibersol**	10	1464	53							1		**1**					
AC	135	18,981	22							1		**1**					
Santos	12	2783	50														
Blau	5	2250	30														0
**Average**	**20,06**	**3720,19**	**32,19**				**0.44**					**2.31**					

Hotel chains in bold had environmental information on their websites and environmental reports, while the rest only had websites. (2) For information about business groups, we only used data from hotel chains

This analysis by group shows us that there is a lot of room for improvement in the EBM of HCs. Most of the groups do not have EBM or have to improve some of their components, especially the value capture component, which is the least developed. Moreover, this room for improvement is also present among the nine HCs that have well-developed EBM, especially the value proposition component, where its mean (9) is far from the maximum value of this variable (17).

## Conclusions and limitations

### Theoretical implications

This study contributes in two major ways. First, it contributes to the literature on environmental management in HCs by proposing EBMs as a useful tool to integrate all the aspects that must be taken into account in the environmental management of hotels. Many studies and institutions have pointed out the environmental impact of the hotel sector, and researchers have adopted different perspectives. However, although these studies have been useful to advancing knowledge, their partial perspectives can provoke incomplete diagnoses that may lead to errors in environmental management and public policy decisions. Thus, corresponding to recent literature (Coles et al. 2017), we consider that BMs provide an integrated vision of the set of activities and processes carried out by firms when defining environmental value proposition, creation, and capture for their stakeholders.

Second, this paper identifies the key elements that BMs in the hotel industry must contain in order to show systematic environmental commitment. Thus, EBMs are conceptualized in this industry as the result of a balanced emphasis on several initiatives regarding proposition, creation and capture of environmental value. The literature reviewed a wide range of tourism and manufacturing. Research efforts have made it possible to identify several key elements that conform to the framework of HC EBMs (Table 10). This framework has allowed the study of the current level of development in HC EBMs and the analysis of their main strengths and weaknesses.

**Table 10:** Framework of environmental BM components

Business model component	Environmental value proposition			Environmental value creation		Environmental value capture	
Key elements	Identify stakeholders’ environmental needs	Environmental issues in mission, vision and/or objectives	Environmental-friendly service offers	Resources, processes and practices in relation to environmental management	Partners and contact networks for management environmental issues	Revenue and cost	Environmental performance indicators
Examples of authors	(Bocken et al. 2014; Hogelvold et al. 2015; Laasch 2018)	(Hogelvold et al. 2015; Laasch 2018; Seelos and Mair 2005)	(Bini et al. 2018; Bocken et al. 2014; Laasch 2018; Wells and Nieuwenhuis 2004)	(Bini et al. 2018; Bocken et al. 2014; Hsieh 2012; Mihalic, et al. 2012; Milanés et al. 2014)	(Bini et al. 2018; Bocken et al. 2014)	(Bini et al. 2018; Bocken et al. 2014; Osterwal-der et al. 2005; Richardson 2008)	(Bini et al. 2018; Bocken et al. 2014; Laasch 2018)

### Managerial implications

The test for how this theoretically driven construct has spread in 120 Spanish hotel chains allows us to reach the following conclusions for HC management.

First, a general overview of the results suggests that HC EBMs are poorly developed. Only nine out of 54 HCs have well-developed EBMs; eleven are on the right track; eighteen present poorly developed EBMs because they have to improve two of their components or do not have one of them; meanwhile, the remaining sixteen HCs, present a non-environmental BM, as they have not developed any components for EBMs.

Second, HC EBM components are unevenly developed. Environmental value creation is the most developed component in almost all groups, followed by proposition value and then by value capture. The greater importance of the environmental value creation component compared to the rest of the components could reflect: 1) environmental management with a focus on carrying out initiatives that have mainly a marketing impact (search as an environmental symbolic legitimacy) or a cost reduction; and 2) a partial perspective on environmental management that could be improved through the use of EBMs as an integral environmental management tool. EBMs would help managers as they establish the expressed management of the proposition and capture of environmental value, which are often neglected. Moreover, the EBMs’ overall perspective can help increase the quality and quantity of collected and published environmental information.

Finally, the identification of HCs with a well-developed EBM could help HC managers with low or undeveloped EBMs to design or redesign their EBMs. It is worth noting that an EBM requires intentional design if it is to deliver the expected environmental impact (Bocken et al. 2019). For example, the results show that HCs should incorporate the stakeholders’ environmental needs in their environmental value propositions (in their mission, vision and objectives or in their service offers); in this case the study of the HCs with well-developed EBMs shows “*the materiality analysis of the stakeholders*” (customers, suppliers, employees, administration, shareholders, company, partners and owners, and press and media) as one of their differentiating elements, and this technique can help them to achieve these objectives.

### Limitations

The research has certain limitations which may open up avenues for future studies. First, the study is carried out with information published by the HCs themselves. This can cause some problems such as greenwashing (communicating more environmental actions than those actually carried out) or green-hushing (selectively communicating fewer environmental actions than are practiced) (Font et al. 2017). In this context, there are few HCs that publish environmental value capture information, and financial capture information is not included in most HC websites and reports, so that it could not be checked whether HCs with more-developed EBMs also have the best financial results. However, the data are good enough to evaluate, with prudence, the proposed framework to HC EBMs and to identify the HCs that will be benchmarks to the managers who want to improve or implement EBMs in their HCs.

Second, this study focuses only on the environmental aspect of sustainability. Future studies should also analyse social problems, as some of these may give rise to synergies with environmental management.

## Appendix

Annex 1: Examples of the codification process.

**Table:**

PROPOSITION		
Key elements	**Examples**	**Codification**
Identification of stakeholders’ needs	Melia Hotels (Consolidated Management Report 2018, p. 26): “*The materiality analysis developed has helped to understand the expectations, requirements and issues of relevance identified by stakeholders, allowing the analysis and implementation of different initiatives that also ensure alignment with Sustainable Development Goals, with new requirements in environmental, social and corporate governance material, from both a global and regional point of view.*” (https://www.meliahotelsinternational.com/es/shareholdersAndInvestors/LegalDocs/Policies/Politica_relacion_con_grupos_de_interes_2018_ES.PDF): “*The purpose of this policy, of global scope, is to establish the principles and guidelines that must govern the relations of Meliá Hotels International S.A. and its Group with the different interest groups with which it interacts, understanding as such, the following groups: employees, partners and owners, shareholders and investors, customers, society and local communities, public administrations, press and media, suppliers.*”	This was coded with 8 because it takes into account the needs of eight stakeholders: customers, employees, suppliers, administrators, shareholders, society, partners and the press
Definition of mission, vision and objectives	Fuerte Group Hotels (Corporate Social Responsibility Report 2017, p.11 and 24): Mission: “*Be a family company with a development vocation and orientation towards profitable hotel and real estate businesses and committed to the responsible development of people and their environment.*” Vision: “*Be a company with leading brands in responsible hospitality, understood as an attitude of sensitivity towards people (shareholder, customer and employee satisfaction) and the environment (local community and environment), with a presence in Spain.*” Objective: “*1% reduction of the corporate carbon footprint compared to the previous year*.”	This was coded with 3 because this hotel took into account environmental aspects in the definition of its mission, vision and objectives
Definition of environmentally friendly service offers	(https://www.nh-hoteles.es/corporate/es/compania-responsable-y-sostenible/sostenibilidad/hoteles-sostenibles): “*At NH Hotel Group we design and operate Eco-Efficient and sustainable hotels. Our design and construction teams consider rigorous sustainability standards so that the eco-efficiency variable is included from the initial phase of the project.*” NH Hotel Group (Annual Report 2018, p.54): “*The Company includes in its gastronomic offerings products that are gluten-free, organic, low in sugar and low in trans and saturated fats.*” NH Hotel Group (Annual Report, p.80): “*The Company offers mobility services such as car sharing or bicycle rental*.”	This was coded with 3 because it offers environmentally sustainable accommodation, food and transportation services

CREATION		
Key elements	**Examples**	**Codification**
No. of initiatives	https://www.bluebayresorts.com/index.php?accion=legal&subaccion=medio-ambiente): *“Maintaining equipment and work vehicles in good condition in order to minimize noise pollution and emissions to the atmosphere.”* *“Energy saving systems in rooms, with a magnetic switch that automatically turns off the power, and sensors for automatic shutdown of air conditioning systems in the case of open windows and terraces.”* *“Installing toilets with savings systems and low-flow toilets and saving systems in the dumps.”* *“Program of separation and recycling of solid waste and used oil. Proper treatment of hazardous substances and waste by authorized companies.”* *“Conservation programs for plants, such as mangroves, and wildlife, such as sea turtles.”*	This was coded with 6 because it presents initiatives in energy efficiency, reduction of CO_2_ emissions, water conservation, waste generation, biodiversity protection and reduction of noise pollution
Presence of environmental management systems	Meliá Hotels (Consolidated Management Report 2018, p. 121): *“During 2018 we have consolidated the certification of our energy management system under the criteria of the ISO 50001 standard.”* *“In addition, this year we have started the certification of our environmental management system under the criteria of the ISO 14001 standard.”*	This was coded with 2 because it has two EMs
Presence of ecolabels	NH Hotel Group (Annual Report 2018, p.78 y 79): “*Up to 141 hotels have individual external sustainability certifications and globally recognized BREEAM, LEED, Green Key and Hoteles* + *Verdes. At the end of 2018, NH Hotel Group had 174 hotels with the TripAdvisor Green Leaders distinction*.” *“NH Hotel Group has participated since 2010 in the climate change global sustainability index CDP (Climate Change Project). This independent non-profit organization analyses the environmental performance of companies.”*	This was coded with 6 because it reports that the group has obtained six certifications in sustainable tourism
No. of partners	Hovima Hotels (Sustainability Report 2018, pp. 5–7): *“Approximately 80% of employees have been trained in the knowledge of sustainability and the environment.”* *“Every week, we explain to clients the guidelines to follow in order to recycle correctly.”* *“Every March 24, we join the ‘earth hour’, an initiative organized by WWF.”* *“About 90% of our suppliers are local. We look for committed suppliers, such as Resuenas, which is the first company in the hospitality sector to obtain the STEP certificate. “*	This was coded with 4 because it makes local purchases, provides environmental education to customers and employees, and cooperates with environmental organizations

CAPTURE		
Key elements	**Examples**	**Codification**
CO_2_ emissions	Fuerte Group Hotels (CRS Report 2017, p.50): *“CO*_*2*_* emissions (Kg per guest) 7.05% ↓.“*	This was coded with 3 because it has reduced C0_2_ emissions
Energy consumption	Fuerte Group Hotels (CSR Report 2017, p.50): *“Diesel oil consumption (litres per guest): 0.497 (2016), 0.485 (2017). Propane consumption (Kg per guest): 0.185 (2016), 0.221 (2017).“*	This was coded with 1 because there was an increase in the consumption of diesel and propane
Water consumption	Fuerte Group Hotels (CSR Report 2017, p.50): *“Water consumption (m*^*3*^* per guest) 7.97% ↑.“*	This was coded with 1 because there was an increase in water consumption
Electricity consumption	Fuerte Group Hotels (CSR Report 2017, p.50): *“Electricity consumption (Kg per guest) 0.83% ↓.“*	This was coded with 3 because it reduced electricity consumption
Waste generation	Riu Hotels&Resorts (Sustainability achievements, 2018, p.7): *“Kg per guest night:* 2 kg (2017), 1.6 kg (2018).*“*	This was coded with a 3 because there was a reduction
